# The Genomics of Auditory Function and Disease

**DOI:** 10.1146/annurev-genom-121321-094136

**Published:** 2022-06-06

**Authors:** Shahar Taiber, Kathleen Gwilliam, Ronna Hertzano, Karen B. Avraham

**Affiliations:** 1 Department of Human Molecular Genetics and Biochemistry, Faculty of Medicine, Tel Aviv University, Tel Aviv, Israel;; 2 Department of Otorhinolaryngology–Head and Neck Surgery, University of Maryland School of Medicine, Baltimore, Maryland, USA;; 3 Department of Anatomy and Neurobiology, University of Maryland School of Medicine, Baltimore, Maryland, USA; 4 Institute for Genome Sciences, University of Maryland School of Medicine, Baltimore, Maryland, USA; 5 Sagol School of Neuroscience, Tel Aviv University, Tel Aviv, Israel

**Keywords:** hearing loss, deafness, genomics, epigenetics, gene therapy, regeneration

## Abstract

Current estimates suggest that nearly half a billion people worldwide are affected by hearing loss. Because of the major psychological, social, economic, and health ramifications, considerable efforts have been invested in identifying the genes and molecular pathways involved in hearing loss, whether genetic or environmental, to promote prevention, improve rehabilitation, and develop therapeutics. Genomic sequencing technologies have led to the discovery of genes associated with hearing loss. Studies of the transcriptome and epigenome of the inner ear have characterized key regulators and pathways involved in the development of the inner ear and have paved the way for their use in regenerative medicine. In parallel, the immense preclinical success of using viral vectors for gene delivery in animal models of hearing loss has motivated the industry to work on translating such approaches into the clinic. Here, we review the recent advances in the genomics of auditory function and dysfunction, from patient diagnostics to epigenetics and gene therapy.

## 1. INTRODUCTION

**Figure 1:**
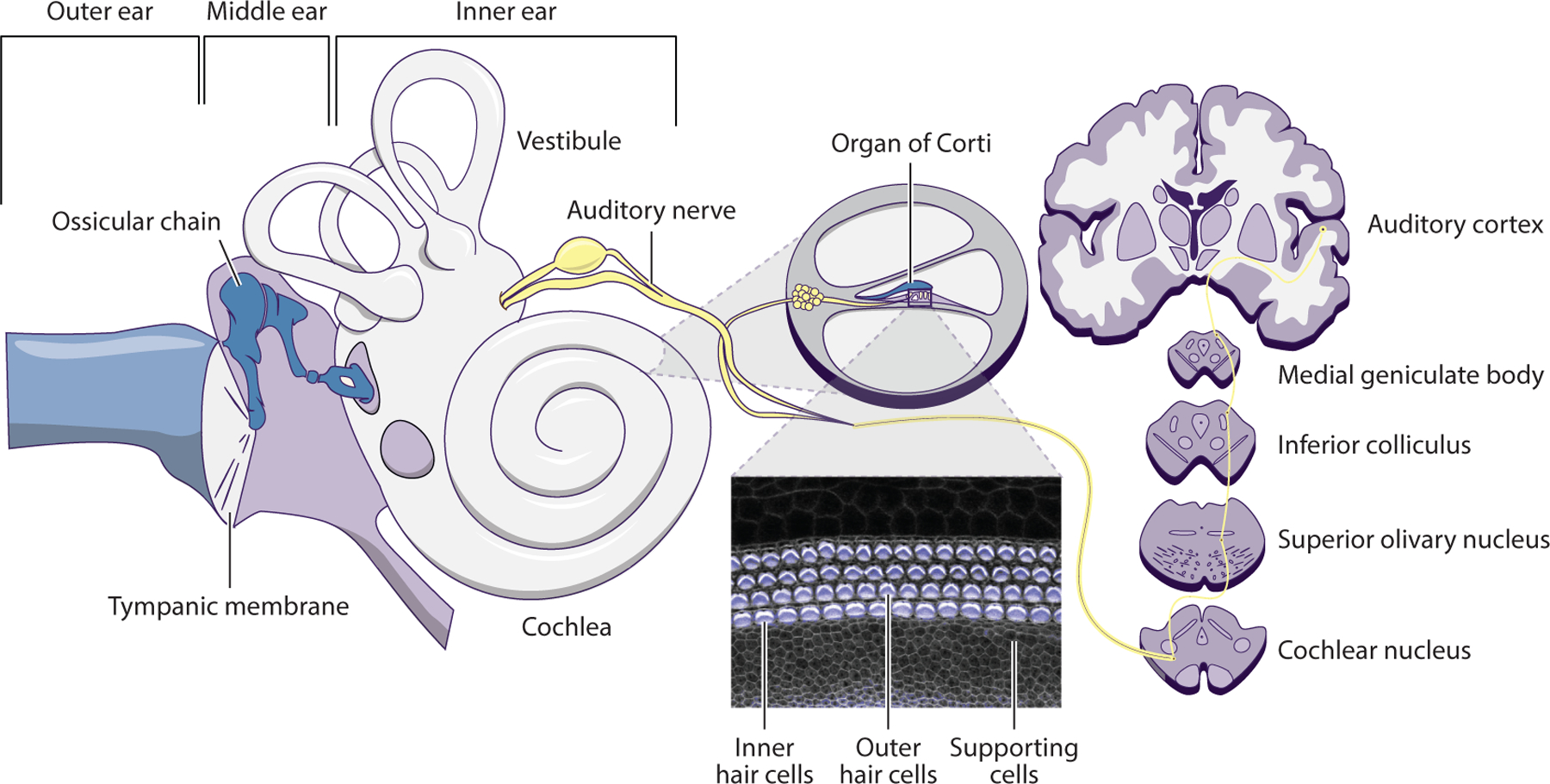
Schematic illustration of the auditory system.

The auditory system is composed of the peripheral auditory system, comprising the outer, middle, and inner ear, and the central auditory system, which is composed primarily of the cochlear nuclei, the superior olivary complex, the inferior colliculi, the medial geniculate body, and the auditory cortex (133). Sound waves transmitted from the environment into the outer ear cause the tympanic membrane to vibrate, and these vibrations are passed on sequentially through the ossicular chain and on to the fluid inside the cochlea in the inner ear (Figure 1). Inside the snail-shaped cochlea, a highly specialized epithelial organ named the organ of Corti is responsible for translating the vibrations into neural signals through a process known as mechanotransduction (45). The organ of Corti houses two types of hair cells: inner hair cells, which are the main receptors of auditory stimuli, and outer hair cells, which function as active amplifiers. Mechanotransduction involves shear-induced bending of the stereocilia on hair cells in the organ of Corti and subsequent depolarization and release of neurotransmitters by the hair cells. Neurotransmitters released by hair cells stimulate the auditory nerve, which then sends the signal up the auditory pathway into the central auditory system. Nonsensory cells in the organ of Corti are referred to collectively as supporting cells. These cells also play integral roles in the function of this organ and are responsible for nutrient supply and ion concentration homeostasis. Hair cells are also found in the vestibule, another structure in the inner ear that regulates balance and spatial orientation.

The intricacy of the inner ear provides multiple possibilities for damage that leads to hearing loss, including environmental accidents, such as prematurity; infections, such as meningitis, rubella, cytomegalovirus, mumps, and chicken pox; head trauma; and ototoxicity (115). Alternatively, since the complexity relies on the correct functioning of highly elaborate pathways, which is dictated by proteins encoded by thousands of genes, a pathogenic variant in any of these genes may lead to hearing loss (137).

## 2. HEREDITARY DEAFNESS

### 2.1. Historical Perspective on Hereditary Deafness Research

Research into hereditary hearing loss began during the late nineteenth century with descriptions of syndromic hearing loss (28). At the time, even Mendel’s laws of heredity were still not widely known or accepted, let alone the chromosome theory, the structure of DNA, the genetic code, and sequencing technologies. Therefore, it was not until the last decades of the twentieth century that deafness was linked to chromosomal regions and then to specific genes. Researchers also identified deafness in mice, the first example of which was possibly the waltzer mouse studied by Rawitz in 1899 (142). The history of deaf mice has been reviewed previously (186); they were also common at the time in Japan, where they were referred to as dancing mice or *Nankin nezumi* (Nanjing mice) for their vestibular dysfunction phenotypes. It is important to note that in the early days, research on hereditary deafness in humans, like research on other hereditary conditions, became part of the eugenics movement in Europe and the United States, a phenomenon that culminated in the sterilization and murder of deaf people in Germany during the 1930s.

**Figure 2:**
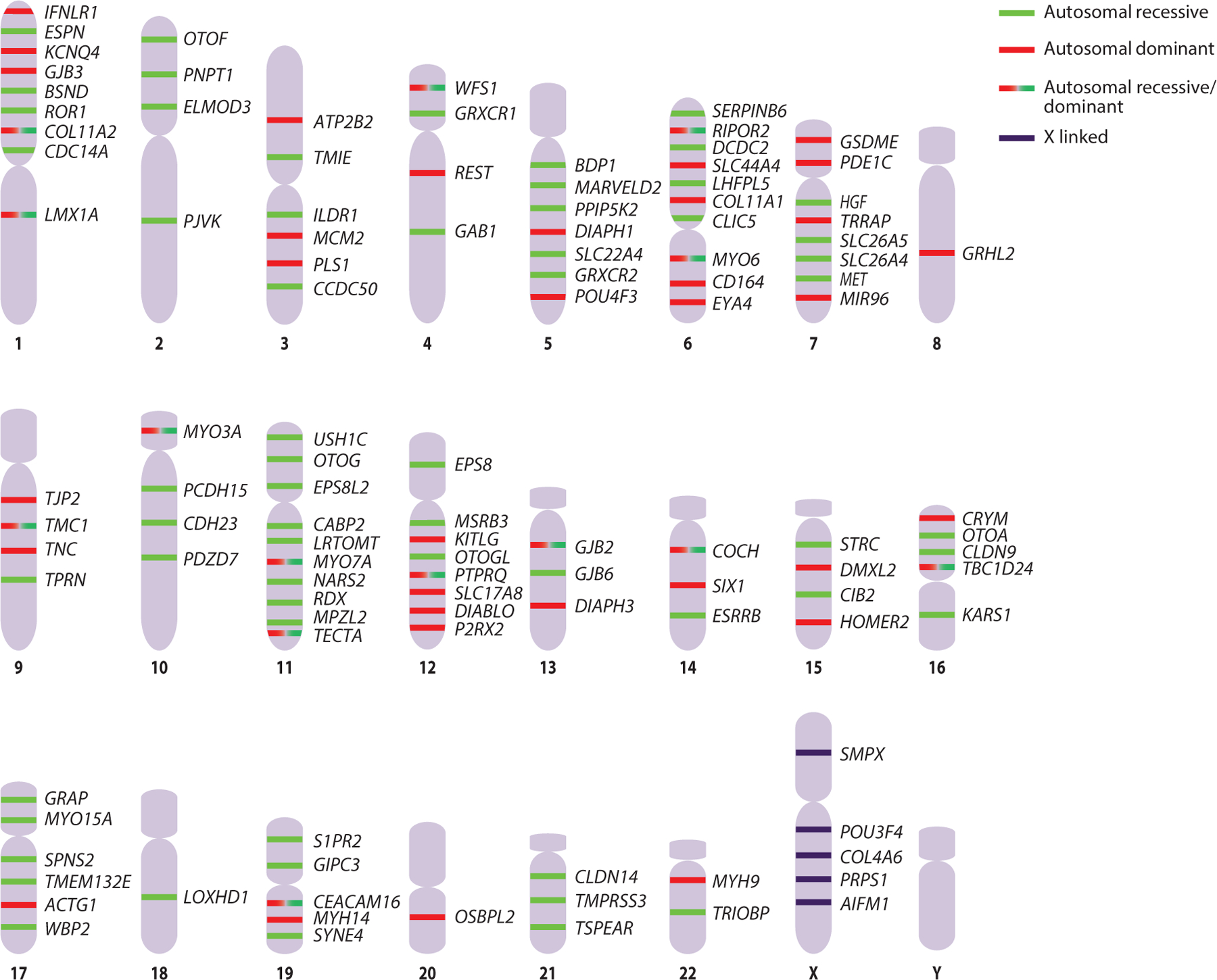
Genes involved in nonsyndromic hearing loss (114) and their positions on human chromosomes.

During the late twentieth century, nonsyndromic hearing loss was associated with chromosomal locations, and the inheritance patterns were termed DFNA for autosomal dominant deafness, DFNB for autosomal recessive deafness, and DFNX for X-linked deafness (Figure 2). This nomenclature is still used today, with the prefix DFNA followed by a number, beginning with the first dominant locus identified in 1995, DFNA1 (96), up to the most recent one, DFNA78 (165). DFNB1 was identified in 1994 (58), and the tally has now reached DFNB108 (165). The first X-linked DFNX locus (later renamed DFNX2) was localized on the X chromosome in 1995 (31) and contained the first deafness gene to be identified, *POU3F4* (30). Soon afterward, a pathogenic variant in *GJB2*, which codes for connexin 26, was identified (78), and this variant is now known to be responsible for the most common form of hereditary deafness (115, 153). Identification of *TMC1* (89), *MYO6* (109), and many more deafness-causing genes soon followed. In 2001, the 13-year, $2.7 billion Human Genome Project was declared complete, with the first drafts of the human genome published (90, 167). The first application in deafness of next-generation sequencing technologies, which emerged during the following decade, was to identify the *TPRN* gene (135), which was followed by rapid growth in the number of genes known to be associated with hearing loss (165).

### 2.2. The Critical Contribution of Deaf and Circling Mice

Research on the genetics of deafness would not be where it is today without the small mammal *Mus musculus*, the house mouse from the Rodentia order. In 1928, a waltzer named shaker-2 appeared, whose mutant gene was subsequently identified by a bacterial artificial chromosome transgene rescue as *Myo15a*, which encodes the myosin XVa protein (128). The study of the mouse mutant and gene made it feasible to identify the human gene for DFNB3 (172), and *MYO15A* mutations were subsequently found worldwide (134). The first molecule associated with auditory function that was identified by genetic mutations was myosin VIIa, which is encoded by the *Myo7a* gene and is responsible for the shaker-1 spontaneous mouse mutant (52). This discovery led to the identification of the human homolog, which is responsible for both nonsyndromic hearing loss and Usher syndrome 1B (177). The next circling mouse mutants to have their genotype elucidated also had mutations in genes encoding myosins, notably the Snell’s waltzer mice, which have a mutation in *Myo6*, which encodes the myosin VI protein (8). This led to the subsequent identification of *MYO6* mutations in an extended Italian family (109) and later throughout the world. *N*-ethyl-*N*-nitrosourea mutants created in several large-scale projects (72) were instrumental in the identification of a significant number of murine deafness genes, many of which led to finding human deafness genes. Prominent examples include the Beethoven model, which is associated with mutations in the *Tmc1* gene (171).

### 2.3. Deafness Today Worldwide

The past decades have seen the gradual introduction of newborn screening programs for deafness in many countries around the world (115). The current estimate of the rate of congenital bilateral hearing loss is 1.33 per 1,000 live births (86, 115). Genetic hearing loss can be categorized into sensory hearing loss, auditory neuropathy spectrum disorder, and central hearing loss, a division based on whether the affected component of the auditory system involves the hair cells, the auditory nerve and/or synapses, or the central auditory system, respectively (86). While in developed countries the primary cause of congenital hearing loss involves genetic variations, in utero or perinatal infections and exposures remain an important risk factor for prelingual hearing loss in low-income countries (86). Cytomegalovirus is a well-known and common cause of congenital hearing loss. Infants infected with cytomegalovirus have a 14% chance of developing hearing loss, and up to a third of the cases will have moderate to profound permanent hearing loss (55). Other potentially damaging infections are rubella and toxoplasmosis. Additional risk factors include perinatal hospitalization and exposure to aminoglycosides (86).

Genetic hearing loss can manifest as congenital, prelingual, or postlingual, depending on the age of onset. There is also heterogeneity with respect to severity, with the American Speech-Language-Hearing Association (ASHA) classification for hearing impairment defined as 25 decibels (dB) or better for no impairment, 26–40 dB for mild impairment, 41–55 dB for moderate impairment, 56–70 dB for moderately severe impairment, 71–90 dB for severe impairment, and 91 dB or greater for profound impairment, referred to as deafness (26). Genetic hearing loss is often further categorized as either syndromic or nonsyndromic hearing loss, based on the presence or absence of abnormalities affecting additional organs and systems other than the auditory system (82). Exome sequencing or targeted sequencing panels typically identify a causative genetic variant in up to 60% of cases (17, 163). Future research on the noncoding genome will hopefully help to increase this rate.

### 2.4. Syndromic Hearing Loss

Approximately 30% of genetic deafness cases are syndromic, where another organ or system is also affected. The best-known forms of syndromic hearing loss are Usher syndrome, which affects both the auditory and visual systems with varying degrees of severity and a range of ages of onset depending on the mutated gene; Pendred syndrome, which is characterized by hearing loss, enlarged vestibular aqueduct, and thyroid dysfunction; and Waardenburg syndrome, which presents with hearing loss and pigmentation defects in various organs, including the skin, eyes, and hair. Syndromic forms of hearing loss further underscore the importance of genetic testing of individuals with hearing loss. A classic example of the potential ramifications of genetic testing in this context is the case of Jervell and Lange-Nielsen syndrome, in which patients present with hearing loss and a long QT interval on an electrocardiogram, which dramatically increases the risk of sudden cardiac death (148). In addition, studying these syndromes in which hearing loss is involved can provide information about the mechanisms of auditory function. Syndromic hearing loss has been reviewed previously in greater detail (82).

### 2.5. Nonsyndromic Hearing Loss

Historically, genomic regions associated with nonsyndromic hearing loss have been classified as described above, according to their mode of inheritance, with DFNA for dominant, DFNB for recessive, and DFNX for X linked. Recessive hearing loss accounts for nearly 80% of such cases. As of August 2021, 124 genes were associated with nonsyndromic hearing loss (165) (Figure 2). Despite this range of options, certain genes and variants are more common than others. Variants in *GJB2*, coding for connexin 26, are responsible for up to 50% of genetic deafness cases in many countries in Europe and in the United States (42, 54, 192). A more restricted example is the p.Ser47Pro variant in TMC1, which is responsible for genetic deafness cases in Moroccan Jews in Israel (16). Presbycusis, or age-related hearing loss, is also considered to have a genetic basis. Rare variants in genes already known to be involved in dominant hearing loss were detected in approximately 25% of individuals with age-related hearing loss versus 7.5% of hearing controls (14).

### 2.6. Newborn Screening and Clinical Implications of Gene Discovery

Many developed countries perform routine newborn screening tests for early detection of hearing loss (150, 175). This screening is typically performed using otoacoustic emissions (OAE) and/or automatic auditory brain stem response (A-ABR). The advantage of this approach is that it is quick, objective, easy to use, and fairly sensitive. False negative results can occur in newborns with progressive hearing loss, where hearing at birth is still unaffected, or in newborns with auditory neuropathy spectrum disorders, in which the outer hair cells are unaffected (175). This is of great importance because it is estimated that 25–50% of deafness cases will not be detected by this approach. On the other hand, false positive results can occur when there is accumulation of fluids in the middle or external ear, and these cases will require further evaluation. Despite these limitations, newborn screening enables early detection of the majority of individuals with congenital hearing loss. The importance of early diagnosis in this context cannot be overstated. Studies have demonstrated that children provided with cochlear implants by the age of 12 months can achieve normal auditory development and language skills (129).

It has been suggested that referral for genetic diagnosis and next-generation sequencing following a pathological newborn screening test might be more cost-effective than further physiological workup, as it can circumvent the need for additional tests required to establish a diagnosis of hearing loss and thereby save precious time (35). In this context, a recent study in China screened 12,778 newborns for 20 hearing loss variants in addition to physiological newborn screening and found that this approach could detect up to 13% more cases than physiological screening alone (174). It is therefore entirely possible that future screening of newborns for hearing loss will gradually become more sequencing based, especially as the cost of sequencing continues to fall. Combining newborn hearing screening and high-throughput diagnostic sequencing is now recommended to optimize early intervention and aural habilitation for hearing-impaired infants (150).

## 3. THE CODING GENOME AND THE INNER EAR

While approaches in forward and reverse genetics still play an instrumental role in illuminating the molecular underpinnings of auditory function, the postgenomic era has also brought transcriptomics, the systematic study of tissue- and cell type–specific gene expression (65, 120). When such studies are coupled with bioinformatic analyses, groups of genes that underlie processes in cell type–specific development/function, aging, or even regulators of these processes can be identified. As transcriptomic analyses require tissue samples, most such studies to date have used tissues from animal models such as mice (71, 84, 100, 145), rats (125), zebrafish (183), or chickens (11). Additional model organisms studied include *Xenopus* (130), bats (103), and guinea pigs (158). Nevertheless, the comparative analysis of cell types from the auditory systems of divergent organisms has proved priceless and has enabled the identification of evolutionarily conserved as well as divergent pathways. Conserved pathways and cell type–specific markers are often used to identify genes with key roles in hearing (182). Conversely, divergent pathways can be utilized to explore the barriers for hair cell regeneration, which—unlike the situation in zebrafish and chickens—does not occur in the mature mammalian cochlea (7).

Early transcriptomic studies of the inner ear focused on the analysis of intact temporal bones or cochlear and vestibular ducts and largely avoided the inner ear due to the small size of the mouse organ and the relatively large quantities of RNA required for analysis (69, 164). Interestingly, a study of gene expression in the inner ear of a human fetus remains one of the few studies of gene expression from healthy human cochleae (136). Improved protocols for recording gene expression from smaller amounts of RNA enabled cell type–specific analyses. Specific cell types can be isolated using flow cytometry, for example, using endogenously expressed fluorescent markers, with or without combining with antibodies for cell surface proteins. Transcriptomic analysis by either microarray or bulk RNA sequencing then follows (39, 67, 68, 104, 145). Such analyses can identify cell type–specific or cell state–specific genes. Bioinformatic comparison of the promoters of cell type–specific genes, in comparison with genes expressed but not specific to the cell type, has proved successful for the identification of key regulators of inner-ear cell type–specific or cell state–specific regulators of gene expression. Relevant examples include identification of the transcription factor ZEB1 as a key regulator of epithelial fate (67), RFX transcription factors as regulators of hair cell terminal differentiation (39), and IKZF2 as a regulator of outer hair cell functional maturation (24). However, the mature inner ear presents a challenge for efficient and atraumatic dissociation, as it comprises a tight sensory epithelium with a robust actin cytoskeleton. Alternative methods, such as immunoprecipitation of tagged ribosomes or analysis of gene expression from nuclear extracts, have been used to circumvent this challenge (24, 68, 106, 162).

The advent and popularization of single-cell transcriptomics as a laboratory technique have revolutionized our understanding and molecular cataloging of the inner-ear cell types. A few examples include the characterization of the developing cochlear sensory epithelium, molecular identification and characterization of the composition of spiral ganglion subtypes, characterization of the composition of the inner-ear lateral wall, analysis of cell types from the vestibular system, and characterization of orthologous cell types in zebrafish and chicken for cross-species comparisons and molecular characterization of hair cell regeneration (11, 19, 75, 84, 85, 102, 108, 126, 127, 152, 156, 179). Approaches vary here as well, from techniques that focus on higher throughput but provide a more limited analysis of the cell type–specific transcriptomes (e.g., 10x Genomics–based methods) to those that analyze fewer cells but at a greater depth or transcript coverage (132, 152).

Transcriptomics also enables the understanding of the molecular underpinnings of disease processes. Here, recent studies include analysis of tissues from vestibular schwannoma (57, 193) and Meniere’s disease patients (46), as well as a detailed cell type–specific delineation of the cochlear response to trauma. One such example is a recent study that generated a cell type–specific blueprint of the molecular response to noise that induces a permanent threshold shift (111). In this study, the investigators were able to intersect molecular changes induced by noise with a database of molecular effects of US Food and Drug Administration–approved drugs, in order to identify candidate therapeutics that can prevent noise- and/or age-related hearing loss. Finally, numerous studies have compared gene expression in animal models with auditory and/or vestibular dysfunction, and the results have shed light on the mechanisms by which genes that are important for hearing or balance mediate essential programs (107, 117, 140, 180).

## 4. THE NONCODING GENOME AND THE INNER EAR

### 4.1. Regulatory Elements

**Figure 3:**
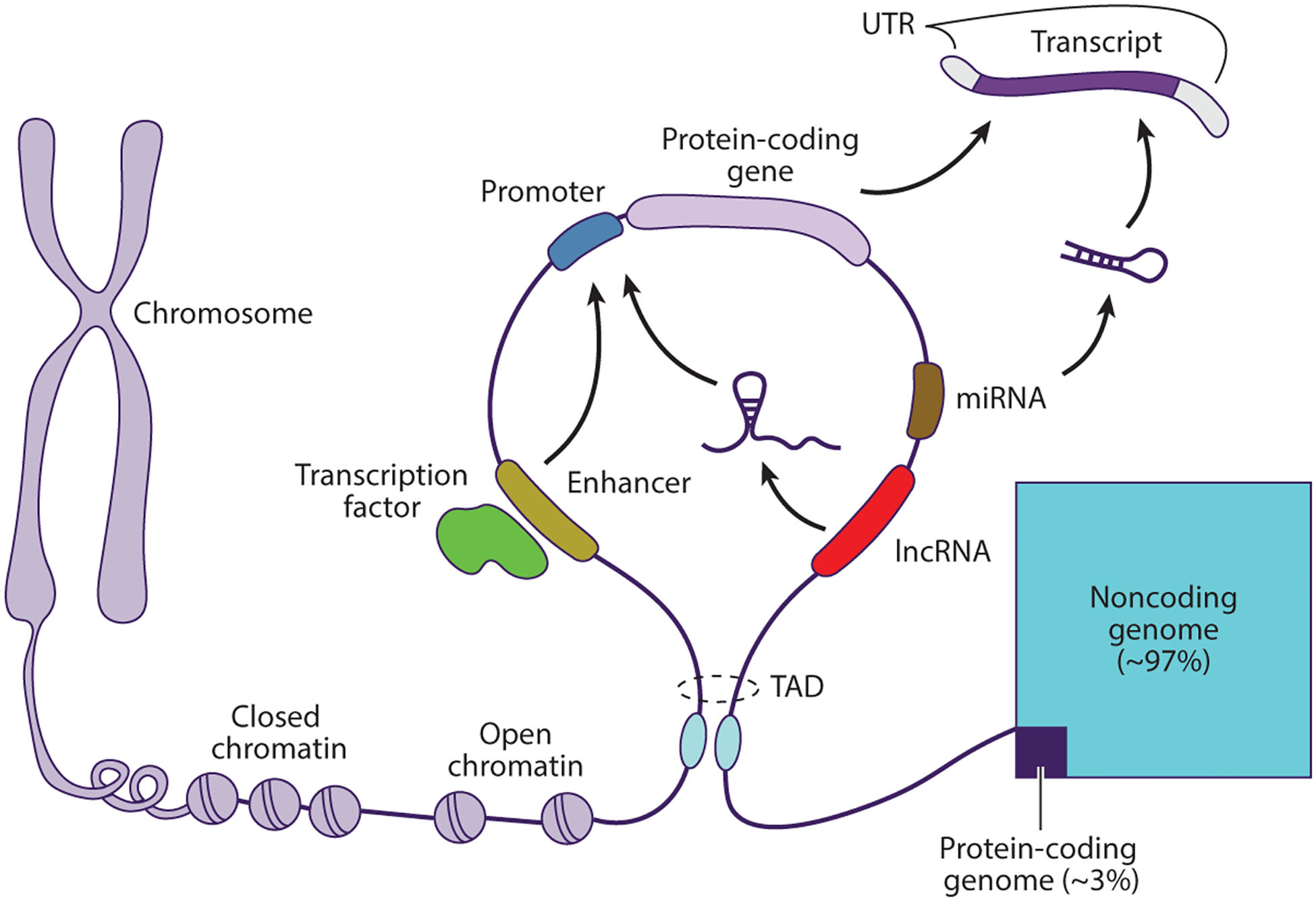
Schematic illustration of genomic structure and the interactions among regulatory elements. Abbreviations: lncRNA, long noncoding RNA; miRNA, microRNA; TAD, topologically associating domain; UTR, untranslated region.

Inter- and intragenic sequences can regulate the temporal and spatial expression of genes, a phenomenon commonly known as epigenetics (Figure 3). Such sequences include promoters, enhancers, repressors, and insulators. DNA-binding proteins, such as RNA polymerase II and transcription factors, interact with specific sequences within regulatory elements to affect gene expression. These sequences can be identified and characterized by studying the biochemical modifications of the DNA molecule and the histones around which it is wrapped. For example, promoters of silenced genes are typically methylated, and lysine 27 in histone H3, where enhancers of actively transcribed genes are found, is typically acetylated (H3K27ac) (81).

With the advent of new genomic technologies, the tools to characterize these elements have become generally available. Bisulfide sequencing technologies have enabled genome-wide profiling of DNA methylation status at single-cell resolution (77). Similarly, chromatin immunoprecipitation followed by sequencing (ChIP-seq) and the recently developed cleavage under targets and release using nuclease (CUT&RUN) and cleavage under targets and tagmentation (CUT&Tag) methods now permit genome-wide characterization of histone modifications and binding of transcription factors (64, 66). Assay for transposase-accessible chromatin with high-throughput sequencing (ATAC-seq) allows the characterization of accessible chromatin regions, which correspond to areas of transcription activity (149). Examining the three-dimensional organization of the genome can facilitate the association between regulatory elements and their target genes by dividing the genome into discrete functional blocks, commonly known as topologically associating domains (139). The Encyclopedia of DNA Elements (ENCODE) and Roadmap Epigenomics projects are two massive collaborative efforts designed to characterize the epigenetic landscape of the human and mouse genomes (12, 41). The results of these projects have identified countless regulatory elements in cell lines and tissues and have made a significant contribution to epigenetics research. Unfortunately, the small size and inaccessibility of the inner ear meant that it was not included in these projects.

This lack has been redressed in recent years, which have seen a growing number of studies that used various technologies to characterize the epigenetic landscape of the inner ear (76, 160, 187). Many of these studies examined the regulation of the developmental programs of the inner ear, in pursuit of the ability to induce hair cell regeneration. For example, the fact that supporting cells in the mouse utricle can transdifferentiate into hair cells more readily than supporting cells in the mature cochlea can be partially explained by the lack of chromatin accessibility in the vicinity of hair cell genes in the latter (76). Studies on regeneration in the inner ear are discussed further in Section 6.3. Characterizing regulatory elements is not only crucial for our understanding of the development of the inner ear but is also likely to prove critical for understanding human deafness, as variants in regulatory elements may well provide an explanation for cases of deafness in which no pathogenic variant has been identified. Proof of principle has been provided by studies demonstrating the importance of specific enhancers for hearing in mice and in humans (10).

### 4.2. MicroRNAs

MicroRNAs (miRNAs) are noncoding RNA molecules with 22–25 nucleotides that were initially described in *Caenorhabditis elegans* and regulate the expression levels of target genes by interacting with mRNAs. Following mature miRNA biogenesis, which includes transcription by RNA polymerase II, cleavage by Drosha and then Dicer, and finally interaction with the RNA-induced silencing complex, the miRNA interacts with its target mRNA by base pairing between the seed region of the miRNA and the 3´ untranslated region of its target gene. This decreases the expression of the target gene as a result of mRNA degradation or translation inhibition. The biogenesis and mechanism of action of miRNAs have been thoroughly reviewed previously (38).

In the inner ear, RNA-sequencing experiments have led to the identification of hundreds of miRNAs (143). Global abolishment of miRNA expression in the mouse inner ear was achieved by conditional knockout of Dicer at different stages of development (47, 73, 154, 166). All such mouse lines exhibit severe cochlear defects, such as thinner cochlear ducts or disorganized stereocilia bundles. The miR-183 family of miRNAs, including miR-96, miR-182, and miR-183, has been extensively studied in this context (144, 178). Mice deficient in any of these miRNAs are deaf and exhibit vestibular dysfunction and stereocilia defects (44, 50, 97, 98). More importantly, two point mutations in miR-96 were found to cause deafness in humans, providing an example of the potential of studying miRNAs in the mouse auditory system (110). In the chicken inner ear, let-7 is important for the precise cessation of proliferation in the developing inner ear (43). Importantly, one of the potential targets of let-7 is Chd7, which is mutated in humans with CHARGE (coloboma, heart defects, atresia choanae, growth retardation, genital abnormalities, and ear abnormalities) syndrome, a condition that includes deafness among its other pathologies (170). Knockdown of let-7a in mice improves survival in a model of graft-versus-host disease, suggesting that exogenous manipulation of miRNAs in the inner ear could be exploited for therapeutic purposes (191).

### 4.3. Long Noncoding RNAs

Long noncoding RNAs (lncRNAs) are a category of noncoding RNA molecules that are more than 200 base pairs long. In every other aspect they behave in the same ways as the mRNAs of protein-coding genes (i.e., performing transcription, splicing, and 5´ capping) (33). Importantly, lncRNAs do not play a direct role in gene regulation, but rather participate in a variety of cellular processes; some lncRNAs actually code for proteins, some are evolutionary remnants of protein-coding genes, and some play a key role in the regulation of gene expression (20, 53, 70). The most studied example of regulation is the case of the X-inactivating transcript (*Xist*) gene (15). It is worth noting that *Xist* is also an example of a lncRNA that is a remnant of a protein-coding gene (37). Hence, the origin of the lncRNAs does not dictate their function or lack thereof. The mechanisms by which *Xist* mediates silencing of the X chromosome are not fully understood and have been thoroughly reviewed recently (15). However, they are believed to include coating of the X chromosome by the *Xist* transcript in *cis* and subsequent interaction with proteins such as SPEN and the Polycomb system, which mediate silencing by means of DNA methylation at CpG islands and modification of histones to produce the repressed state (15).

A handful of studies have characterized the expression profiles of lncRNAs in the inner ear by using high-throughput sequencing technologies (83, 105, 147, 164, 176). Some inner-ear lncRNAs are also expressed in other systems, such as *Malat1*, while others, such as *Xloc_012867*, are novel lncRNAs that are apparently restricted to the inner ear (83). A study identified the lncRNA *Rubie* as a candidate for circling behavior in mice, as it is expressed in the cochlea at embryonic stages and is found in close proximity to Bmp4, potentially regulating it in *cis* (138). Deafness-causing variants in lncRNAs have yet to be discovered in humans, other than an approximately 200-kb deletion that causes deafness and was suggested to result from the disruption of two lncRNAs (99). However, there is evidence that studying the mechanism of action of lncRNAs can lead to therapeutic opportunities regardless of whether they are directly involved in the disease patho-genesis. An example is the case of the lncRNA *Malat1*, which can be knocked down to reduce tumor growth in breast and lung cancer models (5, 60).

## 5. RESOURCES FOR THE SCIENTIFIC COMMUNITY

### 5.1. Deafness Gene Websites

The inner-ear research field is extremely fortunate to have the Hereditary Hearing Loss Homepage, a web-based resource that maintains a list of human deafness genes dating from the early days of gene identification in 1984 (165). It includes the DFN loci and genes associated with syndromic and nonsyndromic hearing loss. In 2011, this resource was joined by the Deafness Variation Database, which describes all of the deafness genes and genetic variations that cause syndromic and nonsyndromic hearing loss (9, 114). These two websites are complementary to one another, and both serve as excellent resources for researchers working in the field.

### 5.2. Gene Expression Analysis Resource

**Figure 4:**
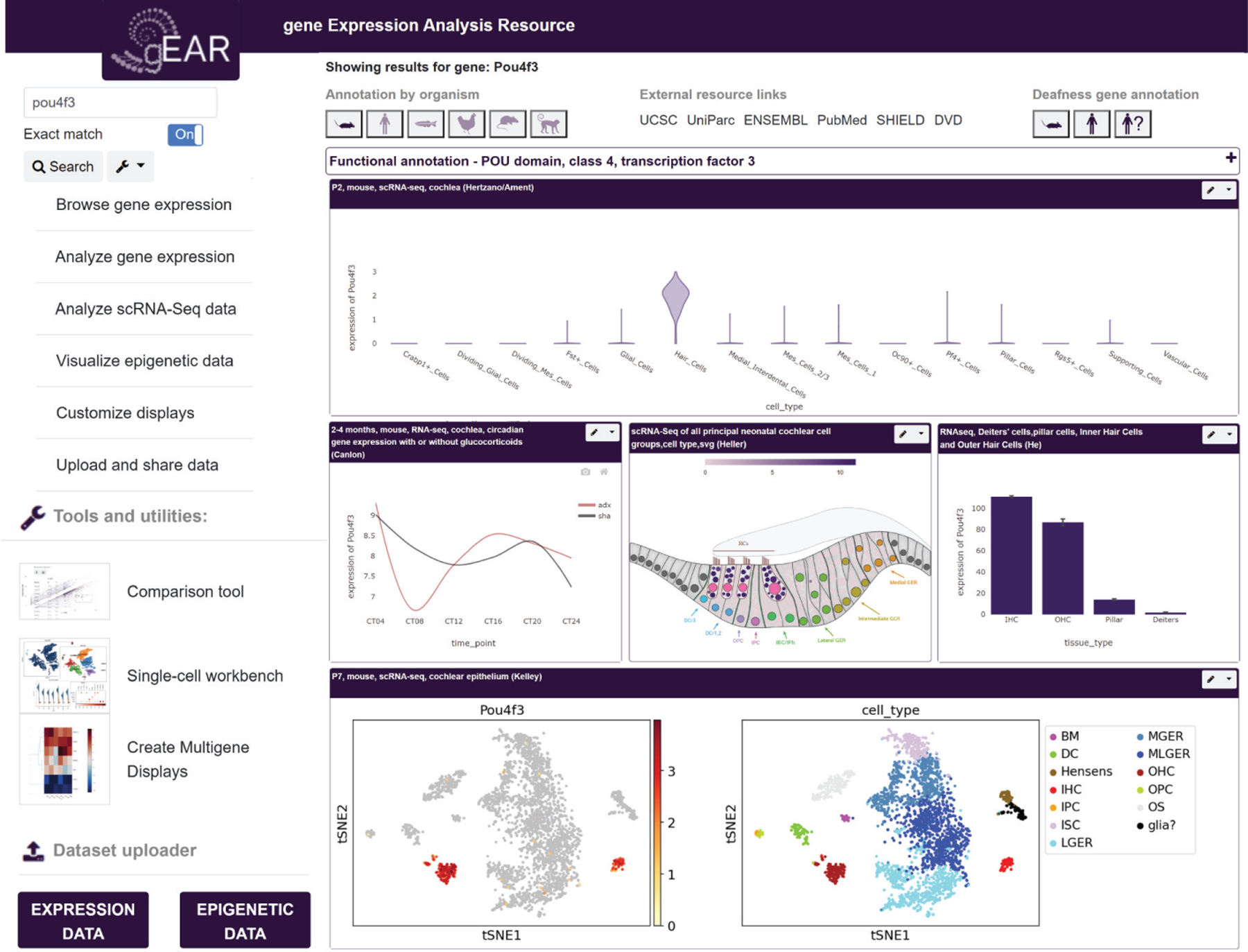
Example of a results page from the gEAR website (https://umgear.org), which provides a centralized portal for sharing, visualization, and analysis of multiomic data in the ear research field. Each data set is represented in a box that allows access to further information and analysis tools, and a variety of data sets and multiomic modalities can be represented on one page. With more than 1,300 registered users and 879 data sets, gEAR has transformed the culture of and access to data sharing in the field. Abbreviation: gEAR, Gene Expression Analysis Resource.

The transcriptomic data described above are usually reported in the peer-reviewed literature and are often accompanied by a table presenting differentially expressed genes. For individuals interested in optimal access to the data, the raw (but often unanalyzed) data are deposited in the National Center for Biotechnology Information’s Gene Expression Omnibus database (27). While consistent with the regulations for data sharing, the file formats are beyond the reach of many researchers, and their download, analysis, and interpretation may be too complicated or time-consuming. Fortunately, the Gene Expression Analysis Resource (gEAR) website (https://umgear.org) provides a centralized portal that aggregates all multiomic data from the ear research field and provides seamless access for data visualization, analysis, and sharing (122) (Figure 4). It has already been used by numerous groups for hypothesis generation, data validation, and dissemination of data, highlighting the importance of meaningful access to transcriptomic and multiomic data.

## 6. THERAPY FOR HEARING LOSS

### 6.1. Gene Therapy

**Figure 5:**
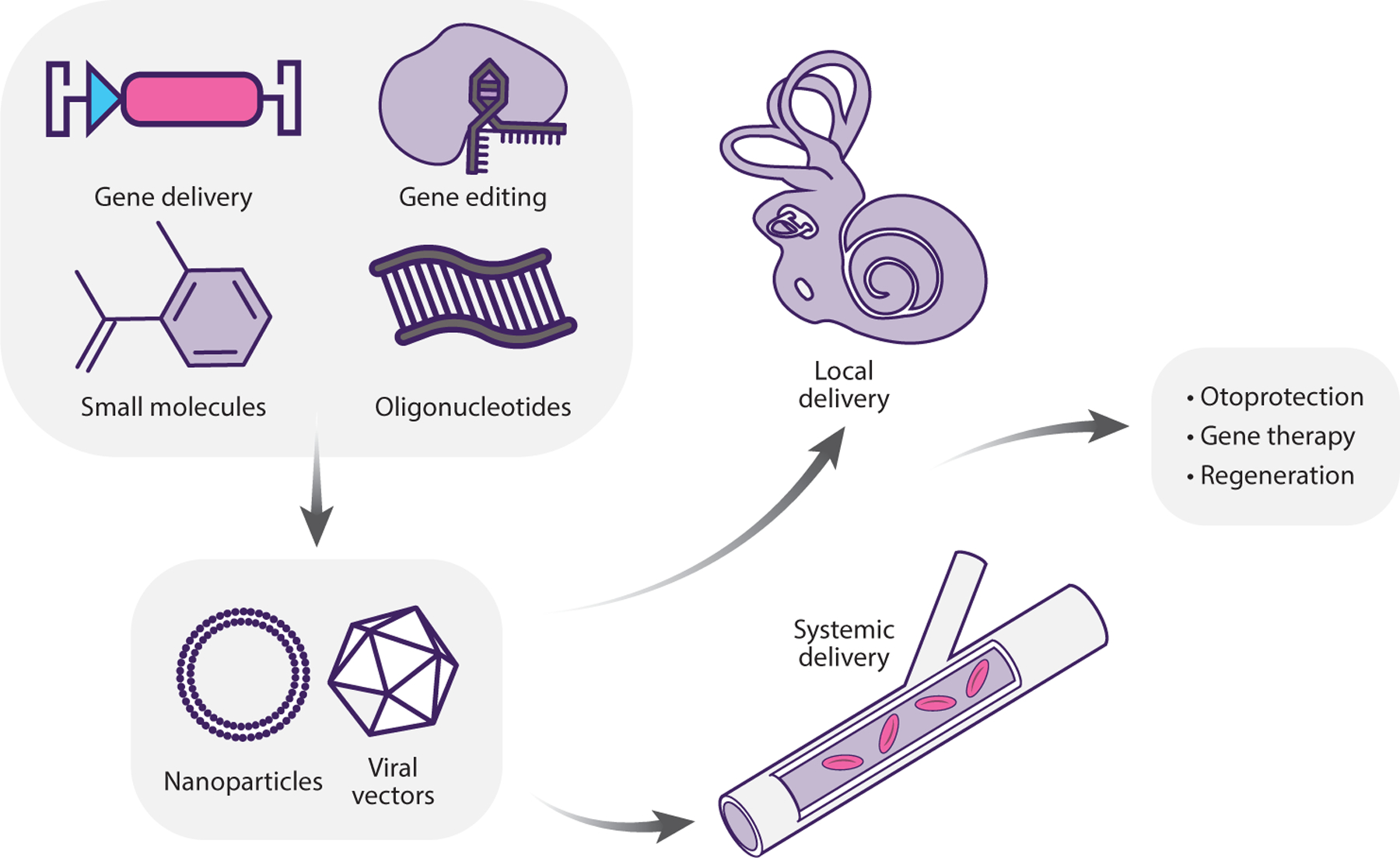
Strategies and modalities of inner-ear therapy.

A natural progression in research on genetic hearing loss (and genetic disease in general) is that the identification of a gene or a genetic variant is followed by the development of targeted therapy. Gene therapy involves the use or manipulation of DNA or RNA for therapeutic purposes (Figure 5). Research into gene therapy for genetic hearing loss is growing rapidly, owing in part to the increasing information available about genes involved in hearing loss and their roles in the function of the auditory system and in part to advances in gene delivery technologies and therapeutic tools (168). Gene delivery in the inner ear most commonly relies on adeno-associated virus (AAV) due to its high efficiency and superior safety profile compared with other viral vectors (168). The popularization of CRISPR-Cas9 gene editing technologies has also had an impact on the inner-ear gene therapy field, and a few key studies are discussed below. Gene editing tools in the inner ear have been recently reviewed (13).

There are a number of strategies for developing gene therapy for genetic hearing loss, with gene replacement by viral vectors being the most straightforward and common approach for loss-of-function variants, where cellular dysfunction is due to lack of a certain protein. Alternatively, adeno-associated virus gene editing may be designed to correct a variant and restore the wild-type sequence. By contrast, some form of gene silencing is preferred for variants that create a dominant-negative effect, where the presence of the mutated protein harms the cell by disrupting the function of the wild-type protein. This may be accomplished either by transcriptional or translational repression or by gene editing. Lastly, alteration of the splicing pattern can be a viable strategy for variants that cause splicing defects. Notably, some types of genetic hearing loss may be treated by more than one strategy, as we discuss further below. The pharmaceutical industry has not ignored these achievements, and numerous companies are currently active in this field (146). Notable genes for which gene therapy has shown preclinical success are discussed below, and Table 1 summarizes the results achieved so far.

#### 6.1.1. *TMC1*.

Transmembrane channel-like protein 1 (TMC1) is a protein expressed by hair cells, which together with additional proteins forms the mechanosensitive ion channel complex (123). As already described, *TMC1* was one of the first genes identified as involved in human deafness and was found to be mutated in several murine deafness models (89, 171). In the Beethoven mouse, a dominant Met412Lys variant creates a dysfunctional protein that leads to deafness. Initially, researchers were able to selectively silence the mutated allele by designing a synthetic miRNA that distinguishes between the two alleles (188). The authors of this study generated AAV vectors carrying this miRNA and injected them into the cochleae of Beethoven mice at postnatal day 15 (P15)–P16 in order to mediate RNA-interference silencing of the mutated allele. Treated mice exhibited slower deterioration of click-evoked auditory brain stem response (ABR) thresholds, but the results were quite modest. A later study described the use of SaCas9-KKH, a CRISPR-based gene editing tool, for selective editing and silencing of the mutated allele (62). This was accomplished by delivering AAV vectors encoding the gene editing complex to the ears of P1 Beethoven mice. The results revealed high editing selectivity for the mutated allele, which resulted in significantly improved ABR and distortion product otoacoustic emission (DPOAE) thresholds at up to 12 weeks of age, together with improved survival of hair cells. Such studies demonstrate the benefits of advancements in gene therapy technologies for functional and histo-logical outcomes.

Deafness associated with variants in *TMC1* can also manifest as recessive. *Tmc1*^−/−^ mice were used as a model of *TMC1* recessive deafness in a study in which the *Tmc1* coding sequence was delivered by injection of AAV at P1 to alleviate hearing loss (181). Treatment improved inner and outer hair cell survival and maintained ABR thresholds of approximately 70–95 dB sound pressure level (SPL), up to 12 weeks of age. Similar results were achieved in the Baringo mouse, another mouse model of recessive *TMC1* deafness caused by a Tyr182Cys point mutation. In another study by the same group, a cytosine base editor, delivered by a dual-AAV system, was used to correct the Baringo mutation to the wild-type sequence and prevent hearing loss (185). Notably, this was the first (and, to date, the only) study in which a recessive variant causing deafness was corrected to the wild-type sequence.

#### 6.1.2. *USH1C*.

The *USH1C* gene codes for the protein harmonin, which is found in the stereocilia of hair cells, where it is thought to function as a scaffold (169). In a knock-in mouse model of human *USH1C* deafness, a c.216G>A mutation creates a splicing defect that leads to the expression of a truncated protein (95). Since this form of deafness manifests as recessive, delivery of the entire coding sequence should be a suitable strategy for these mice (and humans). In a study where the harmonin coding sequence was delivered by injection of AAV into the inner ears of homozygous *Ush1c* c.216G>A mice at P0–P1, ABR thresholds of treated mice were as low as 50 dB SPL at some frequencies, whereas untreated mutants showed no response at the tested intensities (124). An alternative approach in the case of this mouse is to modify the splicing pattern so that the wild-type isoform is expressed. In utero injection of splice-switching antisense oligonucleotides into the developing inner ear at embryonic day 12.5 both rescued hearing and prevented hair cell loss (173). The magnitude of recovery observed was variable, but some mice exhibited hearing thresholds not more than 10 dB SPL higher than those of wild-type mice. Another study that tested intracochlear delivery of antisense oligonucleotides at P1 similarly achieved a significant improvement of hearing sensitivity and prevention of hair cell loss (94).

#### 6.1.3. *OTOF*.

The *OTOF* gene codes for the protein otoferlin, which is involved in vesicle fusion in the ribbon synapse of inner hair cells (141). Variants in *OTOF* cause an autosomal recessive nonsyndromic hearing loss that manifests as profound prelingual deafness (184). Since *OTOF*, together with the necessary regulatory elements, exceeds the packaging capacity of AAV (~4.7 kb), several alternative approaches have been undertaken to circumvent this limitation. Two independent studies used a dual-AAV approach to deliver the *Otof* coding sequence into the inner ears of *Otof*^−/−^ mice (1, 3). Both studies used a *trans*-splicing approach, in which the 5´ and 3´ halves of the coding sequence assemble in vivo and the intervening inverted terminal repeats are spliced out by incorporating artificial splice donor and splice acceptor sequences. Other options that were examined by other groups involved overloading the AAV beyond its capacity or, conversely, delivering a cut-down version of otoferlin (131, 161). Unfortunately, these solutions resulted in little to no recovery of auditory function. Importantly, *Otof*^−/−^ mice injected with the dual AAV-*Otof* at P30, well after the onset of hearing in mice, exhibited wild-type-like ABR thresholds, indicating that *Otof*^−/−^ inner hair cells retain the potential for function for an extended period of time. This finding is compelling because humans are thought to begin to respond to auditory stimuli during the first trimester of pregnancy, and the feasibility of postnatal intervention in many forms of congenital deafness in humans is still not clear (48).

### 6.2. Otoprotection

Otoprotection refers to the protection of the auditory system from external insults such as noise, aging, ototoxins, and drugs. The use of genomic technologies has the potential to elucidate the molecular mechanisms underlying noise- or drug-induced hearing loss and lead to the development of targeted otoprotective therapeutics. For example, the complement pathway has been implicated in noise-induced hearing loss (125). Another report suggested that the mitogen-activated protein kinase (MAPK) pathway is among the earliest responsive pathways in noise-exposed ears (4), while a transcriptome and proteome study implicated the unfolded protein response pathways in aminoglycoside-induced hearing loss (121). Another study found that the expression of up to 3,000 genes is affected following exposure to aminoglycosides, with multiple pathways involved, including the JNK pathway and the nuclear factor κB (NF-κB) pathway (159). Age-related hearing loss suffers from a paucity of genomic studies due to the technical difficulty of obtaining sufficient material from the inner ears of adult animals. One study reported differential expression of 134 lncRNAs in aged compared with young ears, suggesting that future genomic research on presbycusis has the potential to identify targetable pathways involved in this common form of hearing loss (155). Notably, such conditions are generally regarded as multifactorial, with more than one cell type or pathway affected, and it is therefore challenging to pinpoint the fundamental processes that contribute to these pathologies (87, 91).

Information about the molecular pathways involved in common forms of hearing loss is already being translated to therapeutics, as in the example described in Section 3 of intersecting data sets for the transcriptional response to noise with a database of molecular effects of Food and Drug Administration–approved drugs (111). Moreover, recent gene delivery technologies have already been deployed successfully to mitigate drug- or noise-mediated hearing loss in laboratory settings. For example, a recent study reported that AAV-mediated brain-derived neurotrophic factor (BDNF) overexpression in rats can ameliorate the damaging effects of noise on ABR measurements (116). Another study that employed a similar approach described the protective effects of neurotrophin 3 (NT-3) (23), and AAV-mediated NT-4–activity-dependent neurotrophic factor 9 (ADNF-9) overexpression was found to be protective against kanamycin-induced hearing loss (194). Several studies have also shown that overexpression of neurotrophins in the ears of animals deafened by ototoxic drugs promotes enhanced survival and growth of nerve fibers, which could contribute to better clinical outcomes with cochlear implantation (18, 92). Notably, there is a growing body of literature on the use of optogenetics as a way to enhance the precision of cochlear implants, typically involving AAV-mediated delivery of channel rhodopsins prior to implantation of an optic device. A recent review described optogenetic stimulation of the cochlea and its use for restoring hearing (34). As more data accumulate on these forms of deafness and gene delivery and as gene editing technologies become more widely used, more therapeutic opportunities will hopefully emerge.

### 6.3. Regeneration

As the organ of Corti is postmitotic at birth, any insult that results in hair cell loss leads almost invariably to impaired auditory function. For this reason, a vast body of literature has focused on studying the development of the inner ear in the hope of identifying pathways and targets that can be manipulated to induce regeneration of hair cells and recovery of auditory function (56). Two predominant avenues for hair cell regeneration have been studied: the proliferation of supporting cells and their subsequent differentiation into hair cells, and the promotion of supporting cell transdifferentiation into hair cells directly. Broadly speaking, the major challenges in this field are that supporting cells lose their regenerative capacity early in mammalian development and that regenerated hair cells often fail to mature and become functional (56). Early work implicated the Wnt/β-catenin pathway in regulating the proliferation ability of Lgr5+ cells in the cochlea (74), with overexpression of β-catenin in the inner ear leading to the proliferation of Lgr5+ cells and formation of hair cells (21, 151). In addition, inhibition of Notch signaling in supporting cells induces transdifferentiation of supporting cells into hair cells (113). The results of further research have suggested roles for additional signaling pathways, such as the Hedgehog and fibroblast growth factor pathways, in the development and regeneration of hair cells (88, 101).

Recent gene delivery technologies, specifically AAV-mediated gene delivery, have enabled targeted manipulation of transcription factors involved in hair cell differentiation, such as Atoh1 (76). Overexpression of a combination of transcription factors has also been tested and achieved transdifferentiation in adult cochleae (93). Recently, the emergence of genomic technologies has greatly advanced this field of research. A recent report described a feed-forward interaction between Atoh1 and Pou4f3, where Atoh1 induces expression of Pou4f3, which in turn cooperatively enables the activation of further targets of Atoh1 in differentiating hair cells (189). The authors used μATAC-seq and CUT&RUN to show that some targets of Atoh1 are also bound by Pou4f3 and are inaccessible in its absence. These results were then confirmed using viral overexpression. In another study by the same group, the authors used ATAC-seq and CUT&RUN to show that hair cell enhancers bound by Atoh1 are primed at birth and decommissioned in the following week, as evidenced by the enrichment of the histone mark H3K4me1 in enhancers and the subsequent demethylation (160). Importantly, the results demonstrated that inhibition of demethylation by the small molecule glycogen synthase kinase (GSK)–lysine-specific demethylase 1 (LSD1) increases the capacity for transdifferentiation. Genomic research on hair cell regeneration holds great promise for future therapeutics. Research into the use of stem cells in the context of hearing loss has been discussed extensively elsewhere (29).

## 7. CONCLUSIONS

The past decades have seen a revolution in the field of auditory function and hearing loss. A major contribution to this transformation has been provided by the development of genomic sequencing technologies, including exome sequencing, RNA sequencing, and epigenetic profiling technologies such as ATAC-seq and CUT&RUN. These technologies have dramatically increased the number of genetic variants known to be associated with hearing loss and have enabled the identification of key factors and pathways involved in the development of the inner ear and in common forms of hearing loss, thereby opening up avenues for therapeutic interventions. In addition, gene delivery and gene editing tools—primarily AAV and CRISPR-Cas9, respectively—have facilitated the emergence of gene therapy as a new field in the search for hearing solutions. As always, open questions remain regarding the ethical use of genetic data and, in particular, of genetic manipulations. Nonetheless, these scientific strides and the collaborative efforts of scientists in the field to share data and combine forces are currently advancing this field by leaps and bounds.

## Figures and Tables

**Table 1:** Gene therapy results in mouse models

Gene	Variant	Strategy	Delivery	Reference
*Cabp2*	KO	Replacement	AAV-PHP.eB	119
*Clrn1*	KO	Replacement	AAV9-PHP.B	61
	cKO	Replacement	AAV8	36
	cKO	Replacement	AAV8, AAV2	51
*Gjb2*	cKO	Replacement	AAV5	190
	cKO	Replacement	Anc80L65	59
*Gjb6*	shRNA-mediated knockdown	Replacement	Electroporation in utero	112
*Kcnq1*	KO	Replacement	AAV1	22
*Lhfp15*	KO	Replacement	Exo-AAV1	63
*Msrb3*	KO	Replacement	AAV1	79
*Otof*	KO	Replacement	Dual AAV2 quadY-F	1
	KO	Replacement	Dual AAV6	3
	KO	Replacement	Overloaded AAV-PHP.eB	131
	KO	Replacement	Mini-otoferlin delivered by AAV8	161
*Pjvk*	KO	Replacement	AAV8	32
*Slc26a4*	KO	Replacement	In utero AAV1 injection	80
*Syne4*	KO	Replacement	AAV9-PHP.B	157
*Tmc1*	*Tmc1* ^Bth/+^	Silencing by editing	Lipofectamine 2000	49
	*Tmc1* ^Bth/+^	Silencing by RNA interference	AAV9	188
	*Tmc1*^Bth/+^, *Tmc1*^Baringo^, KO	Silencing by editing, replacement	AAV9-PHP.B	181
	*Tmc1*^Bth/+^, KO	Replacement	AAV1	6
	*Tmc1* ^Bth/+^	Silencing by editing	Anc80L65	62
	*Tmc1* ^Baringo^	Repair by editing	Dual Anc80L65	185
*Tmc1* and *Tmc2*	KO	Replacement	Anc80L65	118
*Ush1c*	c.216G>A	Replacement	Anc80L65	124
	c.216G>A	Splicing correction by antisense oligonucleotides	Intraperitoneal or intracochlear injection of antisense oligonucleotides	173
*Ush1g*	KO	Replacement	AAV8	40
*Vglut3*	KO	Replacement	AAV1	2
*Whrn*	*Whrn* ^*wi/wi*^	Replacement	AAV8	25

Abbreviations: AAV, adeno-associated virus; cKO, conditional knockout; KO, knockout; shRNA, short hairpin RNA.

## Abbreviations

AAV: adeno-associated virus
ABR: auditory brain stem response
ATAC-seq: assay for transposase-accessible chromatin with high-throughput sequencing
CUT&RUN: cleavage under targets and release using nuclease
CUT&Tag: cleavage under targets and tagmentation
dB: decibels
gEAR: Gene Expression Analysis Resource
lncRNA: long noncoding RNA
miRNA: microRNA
TAD: topologically associating domain
UTR: untranslated region
